# Mitochondrial genome comparison and phylogenetic position of *Fannia pusio* among the Calyptratae flies

**DOI:** 10.1016/j.heliyon.2024.e27697

**Published:** 2024-03-12

**Authors:** Yesica S. Durango-Manrique, Andrés López-Rubio, Lina A. Gutiérrez, Juan P. Isaza, Giovan F. Gómez

**Affiliations:** a Grupo de investigación Bioforense, Facultad de Derecho y Ciencias Forenses, Tecnológico de Antioquia, Institución Universitaria, Medellín, Colombia; b Grupo Biología de Sistemas, Escuela de Ciencias de La Salud, Facultad de Medicina, Universidad Pontificia Bolivariana, Medellín, Colombia; c Universidad Nacional de Colombia - Sede de La Paz – Dirección Académica, Escuela de Pregrados – Km 9 vía Valledupar – La Paz, La Paz, Colombia

**Keywords:** Illumina sequencing, Colombia, Neotropics, Phylogenomic

## Abstract

*Fannia pusio*, the chicken dung fly species, remains unexplored despite its forensic, sanitary, and veterinary importance in the Nearctic and Neotropical regions. In this study, we obtained the complete mitochondrial genome of *Fannia pusio* for the first time using next-generation sequencing. We compared it with previously published mitogenomes of the genus from the Palearctic region, and its phylogenetic position was studied based on the concatenated protein-coding genes (PCGs) dataset of Calyptratae flies. The circular mitochondrial genome of *F. pusio* is 16,176 bp in length, with a high A + T content (78.3%), whose gene synteny, codon usage analysis, and amino acid frequency are similar to previously reported *Fannia* mitogenomes. All PCGs underwent purifying selection except the *nad2* gene. Interspecific K2P distances of PCGs of *Fannia* yielded an average of 12.4% (8.1%–21.1%). The *Fannia* genus is monophyletic and closely related to Muscidae based on molecular data. Further taxonomic sampling is required to deep into the phylogenetic relationships of the originally proposed species-groups and subgroups within the genus. These results provide a valuable dataset for studying the mitochondrial genome evolution and a resource for the taxonomy and systematics of *Fannia*.

## 1 Introduction

The mitochondrial DNA (mtDNA) is inherited maternally and has a low recombination rate. It has evolutionary rates higher than the nuclear genome and a high copy number per cell, making it a valuable data source for species identification, evolutionary biology, and comparative genomics [1,2]. With the advent of next-generation sequencing (NGS) technologies, rapid data acquisition, lower cost, and time investment than Sanger sequencing have enabled researchers to increase the availability of insect mtDNA genomes [3]. This has led to comparative analyses based on all protein-coding genes (PCGs) rather than just relying on a single or few molecular markers [4].

*Fannia* Robineau-Desvoidy, 1830, is a genus of fly species within the Fanniidae family, encompassing approximately 350 species globally, with 113 documented in the Neotropics [5] and 11 groups and subgroups proposed based on adult morphology [6]. Previous phylogenetic analyses have relied mainly on morphological characters to support the monophyly of *Fannia* [7]. However, a phylogenetic approach based on all PCGs of the mitochondrial genomes and considering representative members of other Calyptratae has yet to be done. Species identification in this genus primarily relies on morphological characteristics of the male genitalia, while identifying females and immature stages is difficult or not always possible [8,9], and molecular reference data remain scarce [10,11].

Mitogenomes play an important role in providing data for evolutionary and phylogenetic analyses and revealing the genetic history of a population [12,13]. Despite the increase of published mitochondrial genomes, only few are available for most Calyptratae flies, particularly the *Fannia* genus [14]. Until now, the mitogenomes of *Fannia scalaris* Fabricius, 1794 (NC_053661) [15], *Fannia canicularis* (Linnaeus, 1761) (NC_068710) [16], and *Fannia armata* (Meigen, 1826) (MT628564) are available from specimens collected in the Palearctic region. However, mitogenomes of species from other biogeographical regions have not yet been published.

*Fannia pusio* (Wiedemann, 1830) has forensic, sanitary, and veterinary importance [[17], [18], [19]] and is widely recorded in the Nearctic and Neotropical regions [20]. Herein, we sequenced, assembled, and annotated the complete mitochondrial genome of *Fannia pusio* and performed comparative analyses with the previously published mitogenomes of the genus from specimens collected in the Palearctic region. In addition, we explored the phylogenetic position of *Fannia pusio* among other calyptrate flies based on all concatenated PCGs dataset.

## 2 Materials and methods

### 2.1 Sample collection and DNA extraction

One *F. pusio* male specimen was field collected in March 2011 in Pajarito, a locality from Medellin, Antioquia, Colombia (06°17′10.7″N, 75°36′43.7″W) at 1929 m above sea level and identified using morphological keys [[21], [22], [23]]. Specimen collection was done under collection permit 16455 issued by CORANTIOQUIA on May 18, 2011. The specimen was preserved in 95% ethanol and stored at −20 °C until DNA extraction. Then, the specimen was dried at room temperature and photographed using a digital camera OPTIKAM Pro 3 connected to a trinocular stereomicroscope (Nikon SMZ745T, Nikon Corp., Tokyo, Japan) as support for species identification. The abdomen was dissected for total genomic DNA using a GenElute™ Mammalian Genomic DNA Miniprep Kit (Sigma-Aldrich) following the manufacturer's protocol. The DNA was quantified using the Qubit dsDNA High Sensitivity assay (Thermo Fisher Scientific, Waltham, MA, USA) and stored at −20 °C until further processing. Species identification was confirmed by comparing *COI* sequences at the barcode of life data systems BOLD and GenBank databases [24,25].

### 2.2 Mitogenome sequencing

The *F. pusio* DNA library was sequenced using an Illumina NovaSeq 6000 (Illumina, Inc., California, US) with 2 × 150 bp pair-end reads, constructed using the TruSeq DNA Nano kit. Reads after trimming were required to have a minimum length of 50 bp. Adapters were removed, and quality was trimmed with Trimmomatic v. 039 [26]. Clean data were de novo assembled using SPAdes v. 3.15.4 with the mode read error correction and assembling with the following parameters: PHRED offset auto-detect, k = 55–77, repeat resolution enabled, mismatch careful mode OFF, mismatch corrector SKIPPED and coverage cutoff OFF [27]. A homology search was implemented to identify the contigs associated with the mitochondrial genome. All contigs were compared to the mitogenome of *F. scalaris* (NC_053661.1) using BLASTn v. 2.13.0 [28] with an e-value of 1e-15. To confirm that the mitogenome was completed, the ends of the identified contig were joined, and the inner region was split randomly, creating an alternative contig, which was used to map the reads with BWA v. 0.7.17 [29] and default parameters. The localization of the mapped reads was visualized in Tablet v. 1.21.02.08 [30].

The assembled mitogenome was annotated using the MITOS web server [31] based on invertebrate mitochondrial genetic code, and annotation curation was performed through comparison with closely related species within the graphical environment of the Geneious Prime software (Biomatters Ltd., v.11.0.15). In addition, PCGs were reconfirmed using NCBI ORFfinder [32], and tRNAs were rechecked using tRNAscan-SE v. 2.0 [33]. The circular map of the complete mitogenome was drawn with Geneious Prime software v.11.0.15 (Biomatters Ltd.).

### 2.3 Comparative analysis among mitogenomes of Fannia

Three species of *Fannia* with available mitogenomes to date (December 12, 2023) were downloaded for comparison with the new *F. pusio* mitogenome (Table 1). The eZmito pipeline was used to calculate and visualize strand, codon, and positional nucleotide biases with the following formulas: AT skew = [A−T]/[A + T], GC skew = [G–C]/[G + C] [34], also to calculate and visualize amino acid and codon usage, and Relative Synonymous Codon Usage (RSCU) across mitogenomes [35]. Genetic distances were calculated using the Kimura 2-parameter (K2P) model between each pair of the 13 core PCGs in MEGA v.11 [36]. Base composition and genome synteny analyses were performed on the Geneious Prime software v.11.0.15 (Biomatters Ltd.). We used DnaSP v. 6.12.03 [37] to calculate the nonsynonymous substitution rate (Ka) and the synonymous substitution rate (Ks), as well as the Ka/Ks ratio for all 13 core PCGs in the *Fannia* mitogenomes. Furthermore, we performed a sliding window analysis of whole mitogenomes using a window size of 200 bp and a step size of 20 bp.

**Table 1:** Mitogenomes of the fly species sampled in this study.

Species	Family	GenBank accession number
*Delia antiqua*	Anthomyiidae	NC_028226
*Fucellia costalis*	Anthomyiidae	NC_042770
*Hylemya nigrimana*	Anthomyiidae	NC_063908
*Lispe assimilis*	Anthomyiidae	NC_058292
*Pegoplata infirma*	Anthomyiidae	NC_050312
*Aldrichina grahami*	Calliphoridae	NC_026996
*Chrysomya albiceps*	Calliphoridae	NC_019631
*Chrysomya rufifacies*	Calliphoridae	NC_019634
*Lucilia cuprina*	Calliphoridae	NC_019573
*Phormia regina*	Calliphoridae	NC_026668
*Chlorops oryzae*	Chloropidae	NC_059894
*Drosophila melanogaster*	Drosophilidae	NC_024511
*Drosophila suzukii*	Drosophilidae	NC_060762
*Drosophila pseudoobscura*	Drosophilidae	NC_046603
*Drosophila curta*	Drosophilidae	NC_060566
*Hirtodrosophila subflavohalterata*	Drosophilidae	NC_070279
*Paraliodrosophila antennata*	Drosophilidae	NC_070278
*Fannia canicularis*	Fanniidae	NC_068710
*Fannia scalaris*	Fanniidae	NC_053661
*Fannia armata*	Fanniidae	MT628564
*Fannia pusio*	Fanniidae	OQ692989^a^
*Melophagus ovinus*	Hippoboscidae	NC_037368
*Ornithomya biloba*	Hippoboscidae	NC_061211
*Graphomya rufitibia*	Muscidae	NC_038210
*Hydrotaea aenescens*	Muscidae	NC_042952
*Hydrotaea chalcogaster*	Muscidae	NC_041089
*Mesembrina meridiana*	Muscidae	NC_063930
*Musca domestica*	Muscidae	NC_024855
*Synthesiomyia nudiseta*	Muscidae	NC_042953
*Azelia* sp.	Muscidae	KP901269^b^
*Nycteribia parvula*	Nycteribiidae	NC_068095
*Phthiridium szechuanum*	Nycteribiidae	NC_068222
*Cephalopina titillator*	Oestridae	NC_046479
*Dermatobia hominis*	Oestridae	NC_006378
*Gasterophilus intestinalis*	Oestridae	NC_029834
*Gyrostigma rhinocerontis*	Oestridae	NC_042379
*Hypoderma lineatum*	Oestridae	NC_013932
*Oestrus ovis*	Oestridae	NC_059851
*Rhinoestrus usbekistanicus*	Oestridae	NC_045882
*Pollenia pediculata*	Polleniidae	NC_053684
*Blaesoxipha lapidosa*	Sarcophagidae	NC_063664
*Miltogramma oestracea*	Sarcophagidae	NC_059872
*Oxysarcodexia thornax*	Sarcophagidae	NC_041072
*Peckia collusor*	Sarcophagidae	NC_041079
*Peckia resona*	Sarcophagidae	NC_041077
*Ravinia pernix*	Sarcophagidae	NC_026196
*Sarcophaga diminuta*	Sarcophagidae	NC_053674
*Sarcophaga josephi*	Sarcophagidae	NC_053666
*Sarcophaga pauciseta*	Sarcophagidae	NC_053729
*Sarcophaga schuetzei*	Sarcophagidae	NC_053681
*Sarcophaga tuberosa*	Sarcophagidae	NC_047405
*Taxigramma karakulensis*	Sarcophagidae	NC_069629
*Paradyschiria parvula*	Streblidae	NC_044702
*Paratrichobius longicrus*	Streblidae	NC_044652
*Clemelis pullata*	Tachinidae	NC_039963
*Ectophasia rotundiventris*	Tachinidae	NC_050938
*Exorista civilis*	Tachinidae	NC_039824
*Lydina aenea*	Tachinidae	NC_063609
*Peleteria iavana*	Tachinidae	NC_063086
*Winthemia sumatrana*	Tachinidae	NC_065138

a Mitogenome obtained in this study.

b The species was originally identified as *Euryomma* sp. but later assigned as *Azelia* sp., a member of the Muscidae family (Grzywacz et al., 2021b).

### 2.4 Phylogenetic analysis

Sixty complete or nearly complete mitochondrial genomes of representative species of the subsection Calyptratae (Diptera: Schizophora), including *Fannia pusio* with some Drosophilidae species and *Chlorops oryzae* (Diptera: Choloropidae) as outgroups (Table 1), were used for phylogenetic analyses. Entire mitogenome records were downloaded using NCBI Batch Entrez (https://www.ncbi.nlm.nih.gov/sites/batchentrez). Then, eZsplit and eZpipe were used to prepare the PCG files [35]. Each PCG was manually aligned, checked, and corrected in Geneious Prime software v.11.0.15 (Biomatters Ltd.) for quality control. A concatenated dataset from the PCGs was obtained to assess the phylogenetic relationships under probabilistic methods, Maximum Likelihood (ML) and Bayesian Inference (BI). We analyzed PCG matrices, including nucleotides in all three codon positions (PCG123) and the first and second codon positions of the protein codon genes (PCG12). Additional phylogenetic analyses were not feasible due to the unavailability of complete mitochondrial genomes for all the included taxa (i.e., rrnL, rrnS).

We used the best-fit model (GTR + G + I) determined with jModelTest2 v. 2.1.10 [38,39] for the ML analysis using IQ-TREE v.1.6.1 [40] with a combination of rapid hill-climbing and stochastic perturbation methods and 1000 bootstrap replicates.

BI analyses were performed using PhyloBayes MPI v. 1.9 [41] using the site-heterogeneous mixture model CAT + GTR, a model more suitable for larger multigene alignments to avoid phylogenetic biases [42]. In each analysis, two independent Markov Chain Monte Carlo (MCMC) chains were run after the removal of constant sites from the alignment and were stopped after the two runs had satisfactorily converged (maxdiff <0.1). A consensus tree was computed from the remaining trees combined from 2 runs after each run's initial 25% trees were discarded as burn-in.

All phylogenetic analyses were conducted on the CIPRES Science Gateway [43] in the High-Performance Computing Cluster at the University of Kentucky Analytics and Technologies. The resulting phylogenetic trees were visualized in iTOL v. 6.7.1 [44] and final editing was performed with Inkscape v.1.3.

## 3 Results and discussion

Overall, 7987207 reads were obtained during the sequencing. After the genome assembly, 603705 contigs were assembled with an N50 of 310 nt, an average coverage of 95.2X and a median coverage of 1.5X. The homology search identified only one contig with 88% identity to the *F. scalaris* genome and an e-value of 0. This contig exhibited a coverage of 225X and a length of 16443 nt. The mitogenome completeness analysis allowed us to detect an artefactual repetitive sequence produced during the assembly at both ends of the contig. After a trim, join, and read mapping strategy, the ends of the contigs were corrected.

### 3.1 Genome size

**Fig. 1:**
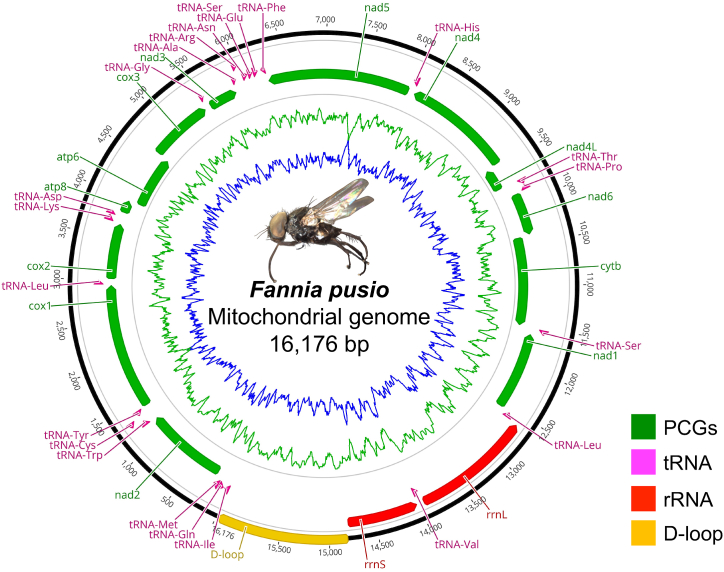
Gene map of the complete mitochondrial genome of *Fannia pusio*. Arrows indicate the direction of gene transcription. Gene names are abbreviated as follows: nad for NADH dehydrogenase subunits 1–6 and 4l; cytb for cytochrome *b*; cox for cytochrome oxidase subunits 1–3; atp6 and atp8 for ATP synthase subunits; rrnL and rrnS for large and small rRNA subunits; tRNA genes are indicated; D-loop for control region. The GC content was plotted using a green sliding window and the AT content was blue.

The circular mitochondrial genome of *F. pusio* is 16176 bp in length. It contains 13 PCGs, 22 tRNA genes, two rRNA genes, and an A + T-rich region (89.3%), also known as D-loop, with a length of 1365 bp (Fig. 1). The nucleotide composition of the whole mitogenome is A 40.1%, C 12.6%, G 9%, and T 38.3%, with a high AT content (78.3%), like other members in the genus: *F. scalaris* (77.9%), *F. canicularis* (79.3%), and *F. armata* (77.8%). Similar results have been found in other insect mitochondrial genomes [45]. In addition, the PCGs in the mitochondrial genome of *Fannia pusio* are typical of insect mitogenomes in terms of length and topology [46].

### 3.2 Gene synteny analysis and tRNAs

**Fig. 2:**
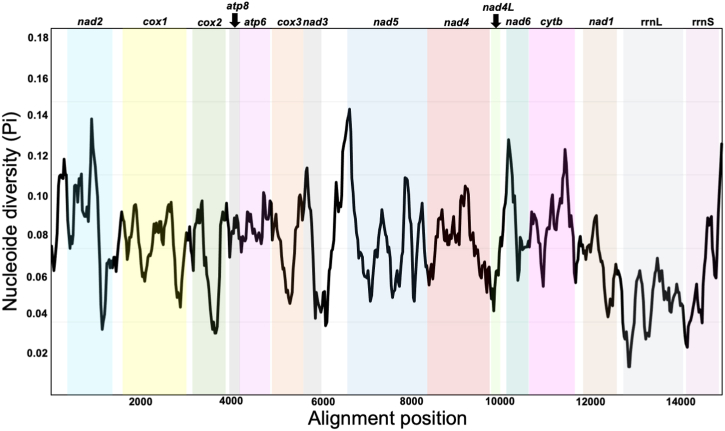
Nucleotide diversity (Pi) among the *Fannia* mitogenomes. The Pi values were calculated from a sliding window analysis of 200 bp in 25 bp steps, represented on the y-axis. The length values of the aligned sequence are represented on the x-axis. The limits of each PCG or rRNA are indicated by coloured bars.

The four mitogenomes from *Fannia* encode an essential set of conserved genes, including three cytochrome *c* oxidase subunits (*cox1, cox2, cox3*), cytochrome *b* (*cob*), two ATP synthase subunits (*atp6, atp8*), seven subunits of NADH dehydrogenase (*nad1, nad2, nad3, nad4, nad4L, nad5, nad6*), and the small and large ribosomal RNA subunits (*rrnS, rrnL*). Comparison among members of the genus evidenced a highly conserved gene synteny among its mitogenomes [16,47] and other dipterans [48,49]. The highest nucleotide diversity values were detected for the *nad2*, *nad5* and *nad6* genes (Fig. 2). Previous studies in insect mitogenomes regarding to phylogenetic signal, highlight *nad2* among the best genes for constructing a tree topology, while *nad5 and nad6* are the best genes contributing to the branch lengths [50].

In the new *F. pusio* mitogenome gene overlap exists between *trnW/nad2* (2 bp), *trnW/trnC* (8 bp), *trnY/cox1* (2 bp), *cox1*/trnL(4 bp), *atp8/atp6* (7 bp), *atp6/cox3* (1 bp), *trnA/trnR* (1 bp), *nad4/nad4L* (1 bp), *nad6/cytb* (2 bp), *cob/trnS* (2 bp) and *trnV/*rrnS (2 bp). Small non-coding regions are mostly 1–6 bp, with the longest between *trnS2/nad1* (16 bp) and *trnE/trnF* (20 bp). Nine PCGs are transcribed on the majority strand (J-strand), whereas four are oriented on the minority strand (N-strand). On the other hand, it has 22 tRNA genes, ranging in size from 63 bp to 72 bp. J strand has 14 tRNAs, and the N-strand has 8 tRNAs. There is one tRNA for each amino acid except Leucine and Serine, which are encoded by two tRNAs each. *Fannia* species evidence a conserved arrangement pattern of tRNA genes, identical to other calyptrate flies [15,51].

### 3.3 At and GC skew

**Fig. 3:**
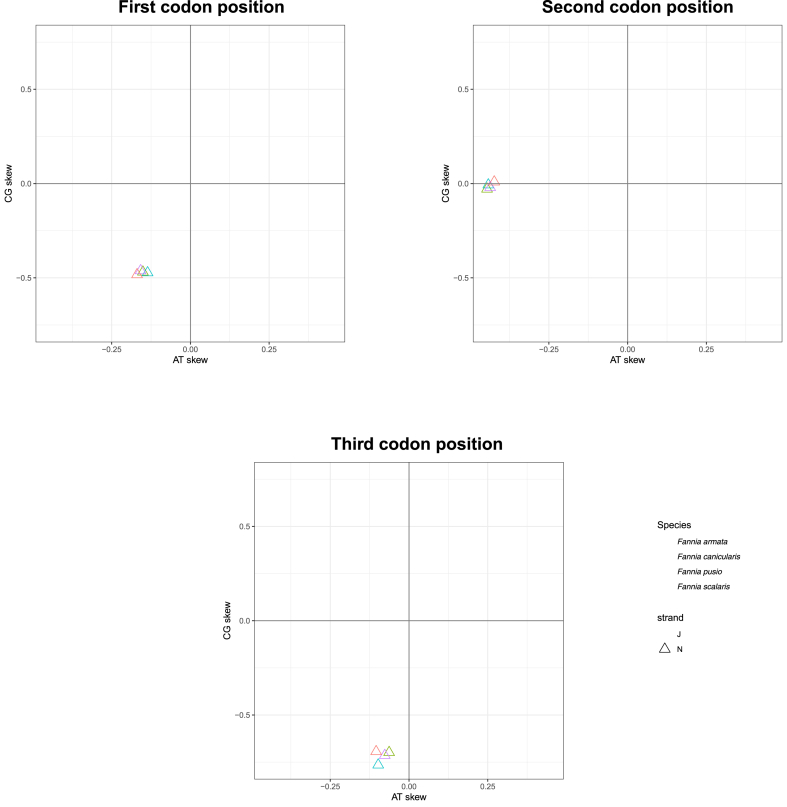
Cytosine **+** Guanine (CG) vs. Adenine + Thymine (AT) skew by codon position with species colour-coded. Strand is indicated using shapes.

The base composition of each codon position for the 13 PCGs shows they all have a high A + T percentage (70.4%–84%). Different codon positions of PCGs show various skew statistics (Fig. 3). The genes on the J-strand showed AT-skew at the first and second codon positions; in the third codon position, all species, except for *F. pusio* and *F. armata*, were AT-skewed. GC-skew was evidenced at the first codon position on the J-strand. The genes on the J-strand had a higher frequency of T (40.9%) and A (34.1%). All species were AT-skewed and GC-skewed on the N-strand for the three codon positions, except for *F. armata* at the second. The genes on the N-strand had a higher frequency of T (48.1%) and A (31.5%). Strand bias in nucleotide composition is concordant with most insect mitochondrial genomes [52].

### 3.4 Interspecific genetic distances among all PCGs

**Fig. 4:**
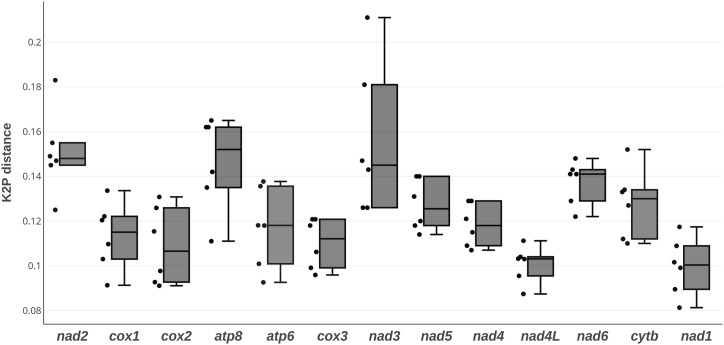
Interspecific pairwise Kimura 2-parameter (K2P) distances of 13 core protein-coding genes among five species of the *Fannia* genus. Box and whisker plot representing the median, upper and lower quartiles, and minimum and maximum values.

Interspecific K2P distances yielded an average of 12.4%, with a minimum of 8.1% for the *nad1* gene between *F. canicularis* and *F. armata*, and a maximum of 21.1% for the *nad3* between *F. armata* and *F. pusio* (Fig. 4). The lowest variance was found for the *nad5* gene, while the highest was detected in the *nad3* gene. The interspecific K2P distance for the *cox1* gene ranged from 9.1% to 13.4%, with an average of 11.3%. Interspecific K2P values for the PCGs are like those found in other flies [[53], [54], [55], [56]]. The success of the *cox1* as a species diagnosis marker and the potential of the other ones requires further sampling to recognize the intraspecific diversity for each PCG [57,58]. Preliminary analyses have shown promising results with the barcode region of the *cox1* gene for species identification of Fanniidae [11,59].

### 3.5 Codon usage analysis and amino acid frequency

**Fig. 5:**
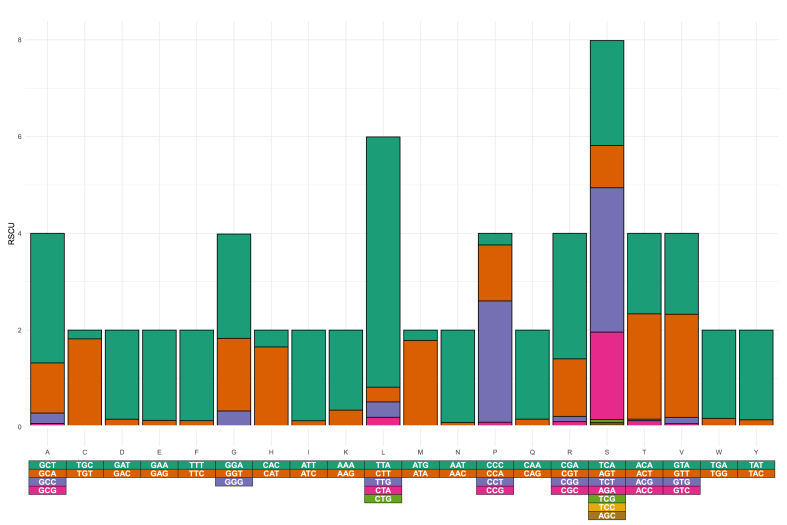
Relative Synonymous Codon Usage (RSCU) of *Fannia pusio*. Codons are color-coded.

**Fig. 6:**
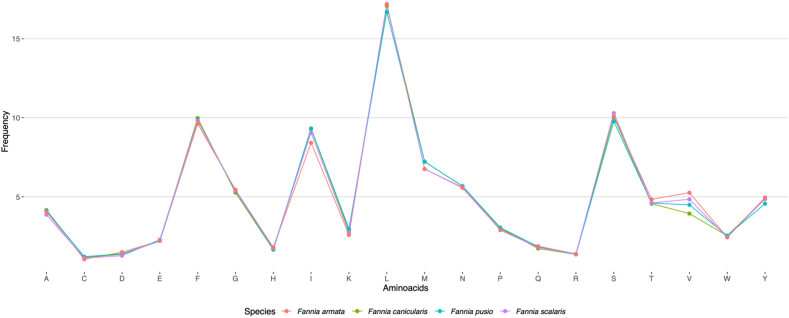
Complete amino acid frequencies by mitogenome. Genomes are color-coded.

A codon usage bias was detected for *F. pusio* codons (52.1% of the RSCU values were higher than 1.0). The most frequently used codons for each amino acid are depicted in Fig. 5. The frequencies of amino acids encoded by the four *Fannia* mitogenomes are highly similar (Fig. 6). Most of the codons encode for non-polar amino acids like Leucine (L), Phenylalanine (F), and Isoleucine (I). In contrast, few codons encode for the polar-uncharged Cysteine (C), the basic Arginine (R), or the Aspartic acid (D), among others. The commonly encoded amino acids for the *Fannia* species mitogenomes are like those found in other insects [[60], [61], [62], [63]].

### 3.6 Evolutionary rates of PCGs

The mean nucleotide frequencies of the PCGs are A = 33.3%, T/U = 43.1%, C = 11.4%, and G = 10.9%, respectively. Among the 13 mitochondrial PCGs, the ratios of Ka/Ks were less than 1 for almost all, except for *nad2*, indicating a strong purifying selection on most PCGs [64]. A higher Ka/Ks ratio in *nad2,* compared to the other PCGs, has been reported in other mitochondrial genomes [[65], [66], [67]], likely related to its small distance from the origin of replication (D-Loop) that exposes it to a higher mutation rate than other PCGs [68], its function [69] or a positive selection in the *nad2* gene [64]. It is an exception, considering that PCGs belonging to complex I region (*nad* genes) have shown a weaker purification pressure compared to PCGs from other mitochondrial complex regions [70].

### 3.7 Phylogenetic analysis

**Fig. 7:**
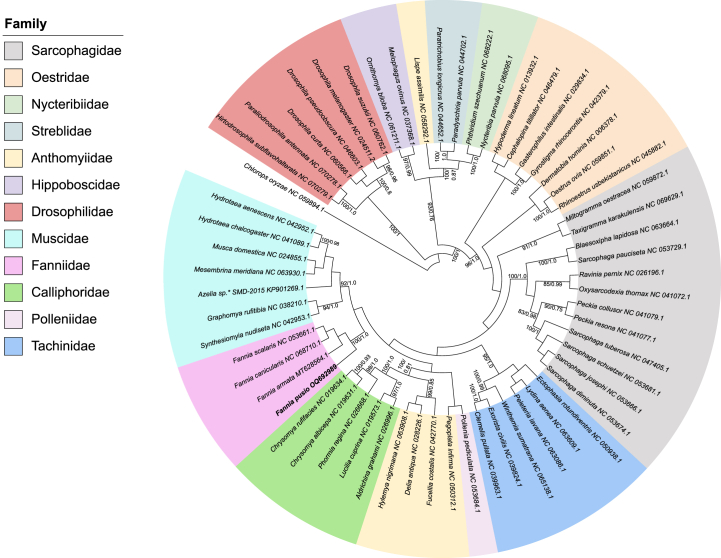
Phylogenetic analysis of *Fannia pusio* among other members of the Calyptratae (Diptera: Schizophora) based on 13 protein-coding genes. The branches show ML bootstrap support (80–100%) and Bayesian posterior probabilities (0.8–1.0).

Both the single gene and concatenated analyses, based on ML and BI approaches, supported the monophyly of the *Fannia* and the position of *F. pusio* as a member of the genus (Fig. 7). Interestingly, including the third codon position (i.e., PCG123 matrix) allowed a better tree resolution than just the PCG12 matrix. Despite third codon positions usually being highly saturated and recommended for exclusion [71,72], our dataset did not reach saturation at this position, adding information for phylogenetic reconstruction. The monophyletic status of *Fannia* based on PCGs of the mitochondrial genome is congruent with previous data based on adult external morphology, female and male terminalia [7], as well as molecular data [73]. Though some authors proposed species groups and subgroups within *Fannia* based on morphological data and a cladistic analysis [7], with the current available molecular data is not possible to test this phylogenetic hypothesis. Thus, we highlight the need for further taxonomic sampling within the *Fannia* genus. Based on morphological data, Fanniidae was initially considered a subfamily of Muscidae during the 1960s (i.e., Fanniinae) [74,75], but later, most specialists recognized it as a separate family within Muscoidea [7,21,[76], [77], [78], [79]]. Herein, the Fanniidae family was closer to the Muscidae, Calliphoridae and Anthomyiidae families, as previously indicated based on a molecular phylogeny of the Calyptratae using mitochondrial and nuclear markers [80] and the musculature of the male terminalia [81].

## 4 Conclusions

The mitochondrial genome of *Fannia pusio* has been sequenced for the first time, revealing a size of 16,176 bp. The gene synteny, codon usage analysis, and amino acid frequency are similar to other *Fannia* species reported previously from the Palearctic region. Phylogenetic analysis supports the monophyly of the genus and its close relationship with the Muscidae family. Further taxonomic sampling is required to delve deep into the phylogenetic hypothesis of the proposed species-groups and subgroups based only on morphology and to study the mitochondrial genome evolution in the *Fannia* genus, particularly in the Neotropics where there is a diversity concentration of *Fannia* compared to other biogeographic regions.

## Data availability

The complete Fannia pusio mitochondrial genome and Illumina raw sequence reads have been deposited in GenBank (OQ692989) and the Sequence Read Archive (BioProject ID: PRJNA1003469, SRR25570796), respectively. Mitogenomes from other Fannia species and others were downloaded from GenBank, with accession numbers and references listed in Table 1.

## Additional information

No additional information is available for this paper.

## CRediT authorship contribution statement

**Yesica S. Durango-Manrique:** Writing – review & editing, Investigation, Data curation, Conceptualization. **Andrés López-Rubio:** Writing – review & editing, Resources, Investigation, Formal analysis. **Lina A. Gutiérrez:** Writing – review & editing, Resources, Methodology, Investigation, Funding acquisition, Conceptualization. **Juan P. Isaza:** Writing – review & editing, Resources, Methodology, Investigation, Formal analysis, Data curation. **Giovan F. Gómez:** Writing – review & editing, Writing – original draft, Validation, Supervision, Resources, Project administration, Methodology, Investigation, Funding acquisition, Formal analysis, Data curation, Conceptualization.

## Declaration of competing interest

The authors declare that they have no known competing financial interests or personal relationships that could have appeared to influence the work reported in this paper.
